# Risk Factors for Neonatal Mortality in Rural Iganga District, Eastern Uganda: A Case Control Study

**DOI:** 10.24248/eahrj.v7i2.730

**Published:** 2023-11-30

**Authors:** Bruce Donald Ndyomugyenyi, Betty Nabukeera, Davis Natukwatsa, Justus Kafunjo Barageine, Dan Kajungu

**Affiliations:** a Makerere University Centre for Health and Population Research, Iganga-Mayuge Health and Demographic Surveillance Site, Kampala, Uganda; b Division of Epidemiology and Biostatistics, Department of Global Health, Faculty of Medicine and Health Sciences, Stellenbosch University, Stellenbosch, South Africa; c Uganda Bureau of Statistics, Kampala, Uganda; d School of Medicine, Makerere University, Kampala, Uganda

## Abstract

**Background:**

Reducing Neonatal Mortality (NM) is vital in decreasing mortality in children below 5 years. Uganda has reported a significant reduction in under 5 and infant mortality over the past decade while NM has stagnated at 27 deaths per 1,000 live births. The NMR of 34 deaths per 1,000 live births in Eastern Uganda is higher than the national rate.

**Objective:**

To determine risk factors for neonatal mortality in rural Iganga district, Eastern Uganda.

**Methods:**

A matched case-control study was conducted between February and July 2019 in Nakigo and Nakalama sub-counties of Iganga district. Cases (n=91) were neonates that died and the controls (n=182) were live neonates at 1 month. Data on maternal, social demographic and neonatal variables were collected from mothers of neonates at household level. Descriptive analysis was performed to determine the profile of study participants. Data was presented as mean (and standard deviation) for continuous variables, and frequencies with percentages for categorical variables. A conditional logistic regression was performed to calculate Odds Ratios and to establish factors that were independently associated with risk of neonatal Mortality.

**Results:**

Giving birth to 5 or more children (AOR=2.88, 95% CI =1.25–6.63), attending less than 4 antenatal care visits (AOR= 2.27, 95% CI= 1.14–5.54), and giving birth to twins (AOR= 6.30, 95% CI=1.24–32.0) were the risk factors for neonatal mortality while delivering from health facilities was protective (AOR= 0.26, 95% CI= 0.12–0.56).

**Conclusion:**

The risk factors for NM are: - giving birth to 5 or more children, attendance of less than 4 antenatal care visits and giving birth to twins. To reduce the risk of NM, the study re-emphasises the need to put more focus on neonatal care during pregnancy and child birth. The study findings can be utilised to determine priorities for reducing the risk of NM in rural settings.

## BACKGROUND

Neonatal Mortality (NM) is defined as any death that occurs in the first 28 days of life.^1^ Reducing NM is vital in decreasing death in children aged below 5 years. In order to further enhance child survival goals among new-borns, the the United Nations (UN) Secretary General (2007-2016), Ban Ki-moon launched the global strategy for Health of Women and Children, which included; Every Woman, Every Child movement in 2010 and the Every New born Action Plan in 2014.^2^ Reducing new-born deaths is one of the bottlenecks to realising the UN global Agenda of Sustainable Development Goal (SDG) 3, target 3.2, which aims at reducing NM to as low as 12 deaths per 1,000 live births and under 5 years old mortality to as low as 25 deaths per 1,000 live births by the year 2030.^2,3^ The global Neonatal Mortality Rate (NMR) fell by 41%, from 31 deaths per 1,000 live births in 2000, to 18 deaths per 1,000 live births in 2017, however, the rate of reduction between the years-2000 to 2017 was slower than that of mortality in children below 5 years (41% compared to 54%).^3^

In developing countries, several studies have reported maternal, social demographic and neonatal factors as frequent contributors to NM^4–7^ including sex of the neonate and gestational age,^8^ birth weight,^9^ education level,^10^ pregnancy complications,^11^ place of delivery^12^ and poor ANC during pregnancy, delivery and post-delivery services.^13,14^

There are nearly 1.2 million new-born deaths that occur in Sub-Saharan Africa (SSA) each year and NM accounts for an increasing proportion of child deaths.^15^ The main causes of NM (neonatal sepsis, birth asphyxia, complications of preterm birth, respiratory distress, low birth weight and congenital anomalies) do not have to result into death since they are not only preventable, but also treatable.^16,17^

Several factors contributing to the high NM in Uganda have also been reported elsewhere in the African Great Lakes region.^18^ These include; sex of the child, non-receipt of tetanus injection, birth order, mother's history of NM, non-attendance of ANC visits, caesarean section delivery, small baby-size and non-usage of contraceptives among others. These factors have also been found to be important determinants of neonatal mortality.^12^

Regular attendance of ANC can significantly avert neonatal deaths. A quality ANC visit necessitates that medically qualified professionals closely monitor and examine mothers and their babies to identify probable poor maternal health conditions such as; infections, anaemia and other complications.^19^ In relation to regular ANC attendance, is a baby's place of delivery. Institutional deliveries increase opportunities for skilled birth-attendance and mothers' access to equipment and essential commodities that are facility based.^19^ This is important in preventing and reducing maternal and neonatal mortality. Sex of the neonate can impact on neonatal outcomes. Male babies have higher mortality risks which are mostly associated with genetic factors.^20^ Other studies further confirm the dangers associated with short birth intervals. Infants born within 2 years of a previous birth are about two times more likely to suffer neonatal death and this can be explained by maternal depletion syndrome and other associated health problems.^21^

The mode of delivery can also influence a neonatal death. Caesarean delivery rates should not exceed 10 to 15 per 100 live births if improved maternal and neonatal outcomes are to be realised.^22^ The increased rate of caesarean section is associated with an increase in morbidity, which calls for a critical need to provide women and caretakers of babies, with information on the risks and benefits associated with caesarean delivery.^22^ In addition, a child's birth weight is an important determinant of survival since it determines the child's vulnerability to the risk of childhood morbidities.^19^ Many births in Uganda, especially in rural settings occur outside of health facilities and this has often resulted into many children not getting weighed at birth, which in turn, increases their risk to childhood illnesses.^19^ Similarly, birth order affects the child's chances of survival. First births are more likely to result into low birth weight babies compared to subsequent outcomes and the likelihood of low birth weight decreases with an increase in birth order.^19^ This may justify why first born children tend to die more during the neonatal period, compared to children born subsequently. Several studies have shown that twins suffer more from low birth weight, preterm delivery and have much higher neonatal mortality compared to singletons.^23,24^ Twins require specialists' health-care in early life, which is usually not available in low resource settings.^24^

In order to achieve goal 3, target 3.2 of the UN global Sustainable Development Goals (SDGs) agenda,^2,3^ reducing the risk of NM is crucial. Uganda's below 5 and infant mortality rates have significantly reduced over the past decade while the Neonatal Mortality Rate (NMR) has stagnated.^19,25^ The NMR of 34 deaths per 1,000 live births^26^ in Eastern Uganda is higher than the country's average of 27 deaths per 1,000 live births.^19^ The aim of this study was to determine risk factors for NM in a rural population of Iganga district, located in Eastern Uganda where the NMR is higher than the national rate. The study will serve to highlight areas for improving new-born care and in turn, reduce the risk of NM.

## MATERIALS AND METHODS

### Study Design

A matched case-control study design was utilised to compare the frequency of known exposure factors between neonates who died and those who survived.

### Study Area

This study was conducted from February to July 2019 in 2 sub-counties of Nakigo and Nakalama in Iganga district, Eastern Uganda. Iganga district is located about 120 kilometres east of Kampala capital city, along the Uganda–Kenya highway. The area is predominantly rural with subsistence agriculture as the major occupation. Sex distribution among residents is almost equal (female=51%), the dominant religion is Islam (53%) and ‘Basoga’ is the main ethnic group.

### Study Population

Nakigo and Nakalama sub-counties have an estimated population of 50,766 individuals. Cases were defined as neonates that died while controls were live neonates at 28 days of life. The inclusion criterion for cases was neonatal deaths that occurred in the study area during the study period. The exclusion criterion for the case and control groups was neonatal deaths which were brought for burial in the study area during the study period.

### Sampling and Selection of Study Participants

We used lists of neonatal deaths and live neonates reported by Village Health Teams (VHTs) to identify cases and controls respectively. All cases (n=91) were included in the study. One hundred and eighty two (182) controls were selected from a list of neonates who were alive at 28 days, born in the same period of the month and same village as the case group. Each case was matched with 2 controls. Mothers of the case and control groups were alive and they all accepted to be interviewed.

### Sample Size Determination and Sampling Techniques

The sample size was determined using Epi-info statistical software. Calculation of the ability of the sample size to detect differences between cases and controls identified that, 91 cases and 182 controls would be sufficient to detect a minimum odds ratio of 2.5; for risk factors and power of 80% with a two-sided 5% level of significance. A sample size of 273 participants was generated for the study.

### Data Collection and Questionnaire

A structured questionnaire was designed and translated in the local language (Lusoga). A team of Research Assistants (RAs) was recruited and trained to perform data collection. These RAs were trained for 4 days in basic research methodology, study objectives and how to administer the questionnaire. The study questionnaire was pre-tested in a neighbouring village for reliability and validity. Pre-testing involved administering the questionnaire to a small set of respondents prior to full-scale survey. RAs collected data for a period of one month, by conducting face-to-face interviews with mothers of the case and control groups. The study supervisor conducted re-interviews on 20% of each of the RA's surveys, and compared the data with what each RA had collected, to verify for correctness and consistency of information. The study supervisor also conducted sit-in-interviews with each of the RAs on a 30% of the surveys to observe the quality of interviews performed.

### Variables

This study considered explanatory variables related to the neonate, the mother, and their social demographic characteristics to determine their association with (NM) which was the outcome variable. A set of risk factors which are variables associated with an increased risk of newborn death was defined to include socio-demographic and maternal variables of the mother's age, marital status, religion, education status, occupation, number of children delivered, number of antenatal visits, place of delivery, mode of delivery, delivery complications, birth interval, exclusive breastfeeding, distance to health facility, HIV testing and having received a Tetanus Toxoid vaccine. Neonatal risk factor variables included; birth order, type of birth outcome, sex of new-born, birth weight, newborn danger signs, gestation age and examining the newborn (Table 1).

**TABLE 1:** Definitions and Categorisation of Variables in the Analysis

Variables	Variable definition and categorisation
Mother age	Mother age was counted in completed years at the time of interview.
Marital status	1=Married, mothers living together with their spouses
	2=Single, mothers not married
	3= Separated/divorced, mothers no longer in marriage union.
Mother religion	1= Muslims, mothers who believe in Islamic faith
	2= Christians, mothers who believe in Jesus Christ and biblical teachings
Education level	1= Not educated, mothers who never attained any level of education
	2= Primary, mothers who attained primary level education
	3= Secondary, mothers who attained secondary level education
	4= Tertiary, mothers who attained post-secondary level education.
Mother occupation	1= Business, mothers engaged in an enterprising, commercial or professional activity
	2= Housewife, a mother whose work is managing her family's home by caring for children and other family members
	3= Peasant farmer, a mother who undertakes small-scale agriculture or farming for a living.
Number of children	Total number of deliveries a woman gave birth to at 28 weeks or more of gestational pregnancy, including the last pregnancy (1=1 child, 2= 2–4 children, 3= 5 children or more)
Antenatal care visits	A mother's total number of antenatal care visits to a health facility during pregnancy (1= 4 visits or more 2= less than 4 visits)
Place of delivery	A mother's choice of where child birth took place (1= Health facility 2= Community including at home or traditional birth attendant's place). Community deliveries also imply the absence of qualified health personnel.
Mode of delivery	1= Normal, giving birth through the vagina
	2= C-Section, a surgical operation for delivering a new-born by cutting through the wall of the mother's abdomen
Birth complications	Any risk to the mother that occurs during birth. Three forms of complications qualified this variable (retained placenta, excessive bleeding and prolonged labour).
	1= Yes for any of the three forms of complications
	2= No to all of the three forms of complications.
Birth interval	The difference between the birth date of a new-born and the birth date of a previous child (1= 3 years or more; 2= 2 years or less)
Exclusive breastfeeding	Feeding the new-born with breast milk only (1= Yes, 2= No)
Birth order	The order a child is born in their family (1=1st; 2= 2nd; 3= 3rd, 4th or 5th; 4= 6th or more)
Birth outcome	1= Singleton, 2= Twin Birth weight 1= Greater or equal to 2.5 kilograms, 2= Less than 2.5 kilograms
Baby danger signs	Signs that sick neonates show as defined by World Health Organisation (1= No, baby did not experience any danger signs 2= Yes, baby experienced danger signs)
Gestation age	The length of time that an embryo grows inside the mother's uterus (1= 36 weeks or more 2=less than 36 weeks)
Distance to health facility	The distance from a mother's home to a health facility (1= Less than 5 kilometres; 2= 5 kilometres or more)
Examining baby	Physical examination of the new-born by a health professional for any signs or problems of complications (1= Yes, baby examined; 2= No, baby not examined)
Sex of the new-born	1= Male,
	2= Female
HIV Testing	1= Yes, mother tested,
	2= No, mother not tested
Tetanus vaccine	1= Yes, mother vaccinated,
	2= No, mother not vaccinated

### Statistical Analysis

The data was double entered in EPIDATA and transferred to Microsoft Excel for preliminary data cleaning and then to STATA 15 for analysis. All records of entered data were double-checked against the corresponding hard-copies, to ensure accuracy and completeness of the data. Descriptive Analysis was performed to determine the social demographic profile of the study participants. Data was presented as mean and standard deviation for continuous variables, and frequencies with percentages for categorical variables. Explanatory variables were tested at bivariate analysis to measure the association between the outcome variable and independent explanatory variables. Variables that were statistically significant at bivariate analysis (with a *p*-value of ≤ 0.05) were considered for multivariable logistic regression analysis to establish their association with risk of neonatal mortality. Crude and Adjusted Odds Ratios (OR) with 95% Confidence Interval (CI) was used to interpret findings of the bivariate and multivariable analysis respectively (Table 4).

### Ethical Considerations

Ethical approval to conduct this study was obtained from Uganda Christian University Research Ethics Committee (UCU REC number: 13-02-600-00090). Research Assistants (RAs) read a written consent script to each respondent, seeking permission to participate in the study. The consent specified the study purpose, risks and benefits; voluntary participation; confidentiality of information and duration of the interview. Each study participant consented by writing the name and signature on the consent form as proof of voluntary participation. Women who could not write were asked to use their thumb print, marked with ink which they appended on the consent form. Young mothers aged below 16 years were counselled on basic health aspects related to neonatal mortality before giving their full consent. They were considered to have sufficient maturity to understand their lived experiences during pregnancy and child birth. The inclusion of women aged 15 to 18 years in this study was part of the study procedure approved by Uganda Christian University Research Ethics Committee.

Hard copies of completed questionnaires and signed informed consent forms were stored in a lockable cabin while electronic records were password protected on a secure server.

## RESULTS

### Social Demographic Profile of Study Participants

A total of 273 mothers of neonates were included in this study. All mothers who lost their new-born within 28 days after births – the neonatal death (n=91) and those with live neonates at 28 days (n=182) participated in the study. This accounted for a 100% response rate. The mean age of mothers was 26 years (SD ± 0.72) and majority were married (83%). The biggest proportion of mothers (58%) was in the age group of 20 to 29 years while 25% were in the age group of 30 to 39 years. Most mothers (57%) attained primary education while 36% attained secondary level of education (Table 2).

**TABLE 2:** Social Demographic Profile of Study Participants

Characteristics	Cases n (%)	Controls n (%)	Total n (%)
Mother age, mean (SD)	26(±0.84)	25(±1.35)	26(±0.72)
Mother age			
15–19	16 (18)	21 (12)	37 (14)
20–29	48 (53)	112 (61)	160 (59)
30–39	24 (26)	45 (25)	69 (25)
40–49	3 (3)	4 (2)	7 (2)
Marital status			
Married	69 (75)	157 (86)	226 (83)
Single	16 (18)	17 (9)	33 (12)
Separated	6 (7)	8 (5)	14 (5)
Religion			
Muslims	58 (64)	114 (63)	172 (63)
Christians	33 (36)	63 (37)	96 (37)
Education status			
Not Educated	2 (2)	2 (1)	4 (1)
Primary	56 (62)	101 (56)	157 (58)
Secondary	30 (33)	69 (38)	99 (36)
Tertiary	3 (3)	105	13 (5)
Mother Occupation			
Business	17 (19)	34 (19)	51 (19)
Housewife	33 (36)	99 (54)	132 (48)
Peasant farmer	41 (45)	49 (27)	90 (33)

Maternal characteristics of study respondents (mothers of neonates), as well as the neonatal characteristics for both the case and control groups are presented in Table 3.

**TABLE 3:** Maternal and Neonatal Characteristics

Characteristics	Cases n (%)	Controls n (%)	Total n (%)
**Maternal characteristics**			
Number of children			
1 child	30 (33)	40 (22)	70 (25)
2–4 children	33 (36)	97 (53)	130 (48)
≥5 children	28 (31)	45 (25)	73 (27)
Antenatal care visits			
≥4 visits	43 (47)	115 (63)	158 (58)
<4 visits	48 (53)	67 (37)	115 (42)
Place of delivery			
Health facility	55 (60)	87 (48)	142 (52)
Community	36 (40)	95 (52)	131 (48)
Birth complications			
Yes	40 (44)	55 (30)	95 (35)
No	51 (56)	127 (70)	178 (65)
Exclusive breastfeeding			
Yes	3 (3)	36 (20)	39 (14)
No	55 (60)	146 (80)	201 (74)
Birth interval			
≤2 years	35 (39)	82 (45)	117 (43)
≥3 years	21 (23)	60 (33)	81 (30)
Mode of delivery			
Normal	83 (91)	166 (91)	249 (91)
C-Section	8 (9)	16 (9)	24 (9)
Tetanus vaccine			
Yes	85 (93)	165 (91)	250 (92)
No	6 (7)	17 (9)	23 (8)
HIV testing			
Yes	88 (97)	179 (98)	267 (98)
No	3 (3)	3 (2)	6 (2)
Distance to facility			
<5 Kilometres	72 (79)	149 (82)	221 (81)
≥5 Kilometres	19 (21)	33 (18)	52 (19)
**Neonatal characteristics**			
Sex of new-born			
Male	41 (45)	78 (43)	119 (44)
Female	50 (55)	104 (57)	154 (56)
Child weight (Mean SD)	3.13(±0.17)	3.3(±0.09)	3.25(±0.08)
Birth weight			
>2.5 Kg	47 (52)	148 (82)	195 (89)
<2.5 Kg	9 (10)	14 (8)	23 (11) Birth
outcome			
Singleton	79 (87)	178 (98)	257 (94)
Twin	12 (13)	4 (2)	16 (6)
Gestation age			
<36 weeks	22 (24)	23 (13)	45 (17)
≥36 weeks	69 (76)	159 (87)	228 (83)
Birth order			
1	15 (17)	42 (23)	57 (21)
2	36 (39)	43 (24)	79 (29)
3	7 (8)	24 (13)	31 (11)
4–5	7 (8)	17 (9)	24 (9)
6	26 (28)	56 (31)	82 (30)
Baby danger signs			
Yes	49 (54)	110 (60)	159 (58)
No	42 (46)	72 (40)	114 (42)
Examining baby			
Yes	75 (82.4)	145 (80)	220 (81)
No	16 (17.6)	37 (20)	53 (19)

### Risk Factors for Neonatal Mortality at Bivariate Level

Peasant mothers were at a higher NM risk compared to mothers engaged in business. Mothers who gave birth to 5 children or more were at a higher NM relative to those who produced 1 child. The odds of NM were also higher in mothers who delivered 2 to 4 children relative to mothers who delivered 1 child. The odds of NM were higher in mothers who experienced birth complications compared to those who did not. The risk of NM was higher in mothers who attended less than 4 antenatal care visits during pregnancy relative to those who attended 4 antenatal care visits or more. The odds of NM were higher in mothers who gave birth from health facilities compared to those who delivered from the community. Mothers who did not exclusively breastfeed their babies were more at risk of experiencing NM compared to those who exclusively breastfed their babies. Additionally, the odds of NM were higher in new-borns delivered as twins relative to new-borns delivered as singletons. New-borns delivered with a birth weight of less than 2.5 kilograms were at a higher risk of dying compared to those delivered with a birth weight of 2.5 kilograms or more (Table 4).

**TABLE 4:** Association between Explanatory variables and Neonatal Mortality

Variable	Cases n%	Controls n%	uOR (95%CI)	P-value	aOR (95%CI)	P-value
Mother Occupation						
Business	17 (19)	34 (39)	1		1	
Housewife	33 (36)	99 (34)	0.61 (0.29–1.24)	0.006	0.48 (0.04–5.29)	0.708
Peasant	41 (45)	49 (27)	1.52 (0.74–3.14)		1.69 (0.18–16.32)	0.634
Mother age						
15–19	16 (18)	21 (11)	1			
20–29	48 (53)	112 (62)	0.56 (0.27–1.17)	0.369		
30–39	24 (26)	45 (25)	0.70 (0.31–1.59)			
40–49	3 (3)	4 (2)	1.31 (0.23–7.38)			
Marital status						
Married	69 (76)	157 (86)	1			
Single	16 (18)	17 (9)	0.13 (0.36–4.42)			
Separated	6 (6)	8 (5)	0.59 (0.20–0.17)	0.096		
Mother religion						
Muslims	58 (64)	114 (63)	1			
Christians	33 (36)	63 (37)	1.54 (0.47–4.99)	0.677		
Education level						
None	2 (2)	2 (1)	1			
Primary	56 (62)	101 (56)	0.55 (0.076–4.044)	0.588		
Secondary	30 (33)	69 (38)	0.43 (0.058–3.232)			
Tertiary	3 (3)	10 (5)	0.30 (0.029–3.134)			
Number of children						
1 child	30 (33)	40 (22)	1		1	
2–4 children	33 (36)	97 (53)	2.20 (1.19–4.08)		2.88 (1.25–6.63)	0.013
≥5 children	28 (31)	45 (25)	1.83 (0.99–3.38)	0.025	2.48 (1.12–5.51)	0.026
Birth complications						
Yes	40 (44)	55 (30)	1			
No	51 (56)	127 (70)	1.81 (1.07–3.05)	0.025	1.56 (0.78–3.11)	0.210
Antenatal care visits						
≥4 visits	43 (47)	115 (64)	1		1	
<4 visits	48 (53)	67 (36)	1.95 (1.17–3.24)	0.001	2.27 (1.14–5.54)	0.019
Place of delivery						
Health facility	55 (60)	87 (48)	1		1	
Community	36 (40)	95 (52)	0.60 (0.36–0.99)	0.049	0.26 (0.12–0.56)	0.001
Exclusive breastfeeding						
Yes	3 (3)	36 (20)	1		1	
No	55 (61)	146 (80)	0.23 (0.07–0.77)	0.001	3.32 (0.86–12.8)	0.082
Birth interval						
≥3 years	21 (23)	60 (33)	1			
≤2 years	35 (39)	82 (45)	0.83 (0.44–1.57)	0.575		
Birth outcome						
Singleton	79 (87)	178 (98)	1		1	
Twins	12 (13)	4 (2)	6.76 (2.11–21.6)	0.001	6.30 (1.2 4–32.0)	0.027
Birth weight						
>2.5 Kilograms	47 (52)	148 (82)	1		1	
<2.5 Kilograms	9 (10)	14 (8)	2.02 (0.82–4.97)	0.001	2.25 (0.73–8.24)	0.148
Sex of neonate						
Male	41 (45)	78 (43)	1			
Female	50 (55)	104 (57)	0.92 (0.56–1.53)	0.758		
Gestation age						
≥36 weeks	69 (76)	159 (87)	1		1	
<36 weeks	22 (24)	23 (13)	0.46 (0.24–0.87)	0.016	1.02 (0.39–2.70)	0.963
Birth order						
1	36 (40)	43 (24)	1			
2	15 (16)	42 (23)	0.43 (0.20–0.89)	0.077		
3–5	20 (22)	27 (15)	0.34 (0.17–0.66)			
6+	20 (22)	27 (15)	0.88 (0.43–1.83)			
Baby danger signs						
Yes	49 (54)	110 (60)	1			
No	42 (46)	72 (40)	0.75 (0.45–1.25)	0.274		
Distance to facility						
<5 Kilometres	72 (79)	149 (82)	1			
≥5 Kilometres	19 (21)	33 (18)	1.23 (0.65–2.31)	0.524		
HIV Testing						
Yes	88 (97)	179 (98)	1			
No	3 (3)	3 (2)	0.33 (0.05–1.99)	0.204		
Tetanus vaccine						
Yes	85 (93)	165 (91)	1			
No	6 (7)	17 (9)	1.37 (0.52–3.64)	0.521		
Examining baby						
Yes	75 (82)	145 (80)	1			
No	16 (18)	37 (20)	0.39 (0.63–2.30)	0.574		
Mode of delivery						
Normal	83 (91)	166 (91)	1			
C-Section	8 (9)	16 (9)	1.00 (0.41–2.44)	0.989		

### Risk Factors for Neonatal Mortality at Multivariable Analysis

Risk factors for Neonatal Mortality (NM) at multivariable analysis were: - Mothers who gave birth to 5 children or more (AOR=2.88, 95% CI=1.25–6.63) were twice likely to experience NM compared to those who delivered one child. Mothers who attended less than 4 ANC visits during pregnancy (AOR= 2.27, 95% CI=1.14–5.54) were 2 times more likely to experience NM compared to those who attended 4 ANC visits or more. Also, neonates delivered as twins (AOR= 6.30, 95% CI=1.24–32.0) were 6 times more at risk of dying compared to those delivered as singletons. Giving birth from health facilities was protective (AOR= 0.26, 95% CI= 0.12–0.56) (Table 4).

## DISCUSSION

Achieving the global Sustainable Development Goal 3, target 3.2^2,3^ entails the identification of risk factors for NM to reduce the NMR. Typical of previous studies that have highlighted a frequent association of maternal, social demographic and neonatal factors with risk of NM,^4–14^ this study determined NM risk-factors related to the new-born, the mother and their social-demographic characteristics. These risk factors include: - giving birth to 5 children or more, attending less than 4 antenatal care visits and giving birth to twins. Giving birth from a health facility was protective.

### Giving Birth to 5 Children or More

This study reports a higher risk of NM among women who delivered 5 children and more. These mothers were twice more likely to experience a neonatal death compared to those who gave birth to 4 children or less. The odds of NM also increased by two-fold for mothers who delivered 2 to 4 children compared to those who delivered 1 child. This was consistent with other studies which reported that, frequent births expose both the mother and new-borns to a high mortality risk because of the likely dangers involved each time a mother gives birth.^27,28^ Some of the reasons for this finding could be that most mothers whose neonates died were more likely to desire producing more children as replacements for those that died. The study results are consistent with results reported by a study conducted in North Tanzania among grand multiparous women with singleton deliveries.^29^ The investigators reported that giving birth to 5 children or more was associated with maternal and perinatal complications including preterm delivery.^29^ In Uganda, further analysis of demographic and health surveys also reported a significant association between multiple births and NM.^25^ A population-based study in Northern Ethiopia which determined NM in a rural setting also highlighted an association between the number of deliveries and NM.^30^ The findings are consistent with those of this study. A similar population-based study that was conducted in Kurdistan province of Iran generated consistent findings, by associating multiple pregnancies with risk of NM.^31^

### Attendance of Less than Four Antenatal Care Visits

Attendance of less than 4 ANC visits during pregnancy was associated with a higher risk of NM. Mothers who attended less than 4 ANC visits were 2 times more likely to experience a neonatal death compared to those who attended 4 visits or more. Several studies have reported similar findings.^26,32^ The reason for this finding could be that mothers who attend less than 4 ANC visits experience limited contact time with health personnel during pregnancy, which increases their chances of experiencing probable health-risks associated with pregnancy. In 2016, a study that investigated the NM and its determinants in the same study population of Eastern Uganda reported that attendance of ANC at least 4 times during pregnancy was independently associated with lower risk of NM.^26^ These findings are consistent with this study's findings. There is further evidence from an analysis of data from 193 surveys conducted in 69 Low and Middle Income Countries (LMICs), which suggests that frequent ANC visits may serve to increase maternal knowledge and positive health-seeking practices among pregnant mothers as well as, offer an opportunity for identifying and managing danger signs and infections respectively.^32^ These results are also in agreement with the global World Health Organization (WHO) guidelines on ANC for a positive pregnancy experience.^33^ WHO recommends that, a pregnant woman should have 8 ANC contacts with the health system during each pregnancy.^33^

Findings of an analytical study that analysed trends and determinants of NM in Uganda using Demographic Health Survey (DHS) data were consistent with findings reported by this study.^25^ In the 2016 DHS, it was reported that, children whose mothers had fewer than the recommended number of ANC visits were associated with higher chances of experiencing NM compared to children whose mothers attended at least 4 ANC visits.^32^ In a related study conducted in Kenya and Uganda in 2017, it was reported that low ANC attendance is associated with risk of NM. This is consistent with this study's findings.^34^

### Giving Birth to Twins

Giving birth to twins was associated with a higher risk of NM. Mothers who gave birth to twins were 6 times more likely to experience a neonatal death compared to those who delivered singletons. The reason for this finding could be that women who are pregnant with twins are more vulnerable to health-dangers associated with pregnancy and child delivery, since they are regarded as high-risk mothers. Also, women who are pregnant with twins require specialised maternal and neonatal care which is usually unavailable in rural settings like Iganga district where this study was conducted. Other studies have reported findings that are consistent with those of this study.^5,23,35^ The findings of a study that compared mortality among twins and singletons in sub-Saharan Africa between the years 1995 and 2014^23^ also reported a higher burden of NM among twin deliveries compared to singleton outcomes; with the ratio of twin versus singleton NM reported to be 5:0.^23^ In agreement with our findings is another multi-country survey that was conducted by WHO in 2018^35^ which reported that, twin pregnancy was associated with considerably higher rates of adverse neonatal and perinatal outcomes.^32^ Our study findings are also consistent with reports from a study that analysed longitudinal and demographic surveillance data in Gambia.^24^

The study reported a significant contribution of twin mortalities to NM; with twin NM 4 times higher than singleton NM.^24^ Another study that analysed secondary data from 60 LMICs also reported a high prevalence of twin neonatal mortality and therefore, emphasised a need for timely identification of twin pregnancies and improvement in accessing caesarean section for twin pregnancies.^36^

### Giving Birth from a Health Facility

Giving birth from a health facility was protective. The protective effect of health-facility delivery on NM requires further study. This finding may reflect the need to improve neonatal health care services in health facilities where mothers seek maternal care.

### Study Limitations

This is a population-based study that determined risk factors for neonatal mortality outside a health-facility setting, where medical investigation data was not collected. This was a limitation to our study. However, this study generated reliable information on risk factors for neonatal mortality in a rural community setting.

In addition, face-to-face interviews with respondents interfered with the emotions of mothers especially those whose neonates died, and this could have biased the nature of responses. Mothers of the case and control groups were interviewed 5 to 6 weeks after a neonatal death occurred, this could have led to recall bias among the study respondents.

## CONCLUSION AND RECOMMENDATIONS

The study identified 3 risk-factors related to the neonate, the mother and their social-demographic characteristics which were independently associated with risk of NM. They include: - giving birth to 5 or more children, attending less than 4 antenatal care visits during pregnancy and giving birth to twins. To reduce the risk of NM, the study re-emphasises the need to put more focus on neonatal care during pregnancy and child birth.

The study was conducted in Eastern Uganda, an area with relatively high level of neonatal mortality in Uganda and thus, the study's findings can be generalised for the bigger national population. The findings can also be utilised to determine priorities for reducing the risk of NM in rural settings.
